# The ComX Quorum Sensing Peptide of *Bacillus subtilis* Affects Biofilm Formation Negatively and Sporulation Positively

**DOI:** 10.3390/microorganisms8081131

**Published:** 2020-07-27

**Authors:** Mihael Špacapan, Tjaša Danevčič, Polonca Štefanic, Michael Porter, Nicola R. Stanley-Wall, Ines Mandic-Mulec

**Affiliations:** 1 Chair of Microbiology, Department of Food Science and Technology, Biotechnical Faculty, University of Ljubljana, Vecna pot 111, 1000 Ljubljana, Slovenia; mihael.spacapan@bf.uni-lj.si (M.Š.); tjasa.danevcic@bf.uni-lj.si (T.D.); polonca.stefanic@bf.uni-lj.si (P.Š.); 2 Division of Molecular Microbiology, School of Life Sciences, University of Dundee, Dundee DD1 5EH, UK; m.porter@dundee.ac.uk (M.P.); N.R.Stanleywall@dundee.ac.uk (N.R.S.-W.)

**Keywords:** biofilm, pellicle, quorum, sporulation, bacillus, signal, communication, heterogeneity, matrix, surfactin

## Abstract

Quorum sensing (QS) is often required for the formation of bacterial biofilms and is a popular target of biofilm control strategies. Previous studies implicate the ComQXPA quorum sensing system of *Bacillus subtilis* as a promoter of biofilm formation. Here, we report that ComX signaling peptide deficient mutants form thicker and more robust pellicle biofilms that contain chains of cells. We confirm that ComX positively affects the transcriptional activity of the P*_epsA_* promoter, which controls the synthesis of the major matrix polysaccharide. In contrast, ComX negatively controls the P*_tapA_* promoter, which drives the production of TasA, a fibrous matrix protein. Overall, the biomass of the mutant biofilm lacking ComX accumulates more monosaccharide and protein content than the wild type. We conclude that this QS phenotype might be due to extended investment into growth rather than spore development. Consistent with this, the ComX deficient mutant shows a delayed activation of the pre-spore specific promoter, P*_spoIIQ_*, and a delayed, more synchronous commitment to sporulation. We conclude that ComX mediated early commitment to sporulation of the wild type slows down biofilm formation and modulates the coexistence of multiple biological states during the early stages of biofilm development.

## 1. Introduction

Biofilms from multicellular collectives encased in the extracellular matrix and represent the default mode of microbial growth in nature [1,2,3]. Quorum sensing (QS) [4], is a microbial communications system that coordinates the cell density-dependent bacterial gene expression, which often regulates biofilm development [5]. *Bacillus subtilis* is the most studied species in the genus Bacillus [6], and has served over the years as an excellent model to study the development of metabolically dormant and heat resistant spores [7] and more recently as a model of biofilms [8,9,10,11], division of labor [12,13,14,15], and a variety of social interactions (reviewed in [16]). This Gram-positive spore former relies on several peptide-based signaling systems and encodes many Phr-Rap signaling peptide-phosphatases pairs and the ComQXPA QS system, which influence sporulation, competence, and biofilm development in *B. subtilis* [16]. The ComQXPA QS system is widespread among Firmicutes [17]. The ComQ isoprenoid transferase processes and activates the ComX signaling peptide. The peptide binds to the membrane-bound histidine kinase, ComP, which phosphorylates ComA [18]. ComA-P directly activates the transcription of its target genes [19,20,21] including the *srfA* operon, responsible for the synthesis of the lipopeptide antibiotic surfactin [22]. Surfactin is a positive regulator of biofilm development and sporulation [23,24]. PhrC, the CSF competence stimulating factor [25], promotes the phosphorylation state of ComA by inhibiting its phosphatase RapC, but its effect on surfactin synthesis is less prominent [26]. Once phosphorylated, ComA-P also increases the transcription of the pleiotropic regulatory gene *degQ* [20,26,27], which increases the DegU phosphorylation rate [28]. DegU-P is ultimately required for the activation of extracellular degradative enzyme production and regulates the biofilm and spore development.

In biofilms, an extracellular matrix composed of polysaccharides (Eps), proteins, and extracellular DNA [2] glues cells together, but the ratios of each matrix constituent differ depending on the specific strain, media, and growth conditions [29]. In *B. subtilis*, the *epsA-O* operon is involved in the production of a critical polysaccharide component of the biofilm matrix [30], which is essential for the development of floating biofilm (pellicle) [31]. TasA, the major matrix protein, encoded by the *tapA-tasA-sipW* operon (hereafter referred to as the *tapA* operon) forms long fibers and gives structural support to floating biofilm [32]. Although TasA is not essential for the formation of floating biofilms, the *tasA* mutant forms less prominent biofilms [33,34]. The molecular regulation of the operons involved in the synthesis of the biofilm matrix components is very complex [35]. Briefly, Spo0A [31], which is controlled by the phosphorelay of multiple histidine kinases [36], initiates biofilm development. Phosphorylated Spo0A (Spo0A-P) activates transcription of the *sinI* operon [37,38]. SinI inhibits SinR [39], which acts as the central transcriptional repressor of the *epsA-O* and *tapA* operons [31,40]. A moderate amount of phosphorylated Spo0A suffices for the activation of the biofilm matrix production operons. However, as the Spo0A-P levels increase, cells commit to sporulation. DegU-P, which is also gradually phosphorylated, also contributes to biofilm formation [41]. DegU indirectly affects the phosphorylation state of Spo0A, shortening the time window of intermediate Spo0A phosphorylation, required to trigger the synthesis of the extracellular matrix [37]. The Spo0A-P levels also positively affects transcription of *bslA* [42], which contributes to biofilm hydrophobicity and influences transcription from a poly-γ-glutamate (*pgs*) operon that only plays a role during the formation of surface adhered biofilms [43].

The ComX QS system affects the transcription of the biofilm matrix operons by increasing surfactin synthesis. Surfactin triggers potassium ion leakage, which positively affects the activity of KinC [23]. This histidine kinase then increases the phosphorylation state of Spo0A [24]. Thus, it has been hypothesized that the ComX QS system increases the phosphorylation of Spo0A indirectly via the lipopeptide surfactin [23]. Consequently, ComX should promote pellicle biofilm formation and may also influence sporulation.

Cells resident in biofilms simultaneously express multiple biological states and this phenotypic heterogeneity results, for example, in the coexistence of matrix producers and highly resistant spores in one biofilm [9,24,44]. QS often regulates adaptive traits that exhibit phenotypic heterogeneity in many different species [45]. However, how ComX density-dependent communication affects the distribution of biological states devoted to the production of matrix components or sporulation in the pellicle biofilm, is less understood.

This work investigates the role of the ComX dependent signaling in pellicle biofilm development and sporulation. It describes the ComX dependent biofilm phenotype and temporal heterogeneity of matrix producers and spores in the pellicle biofilm. Our results provide evidence that ComX positively affects sporulation, but not biofilm development and offers a new outlook on the phenotypic heterogeneity of the phenotypes associated with ComX deficiency.

## 2. Materials and Methods

### 2.1. Bacterial Strains and Strain Construction

All *Bacillus subtilis* strains used in this study are listed in Table 1. The recombinant strains were constructed by transforming specific markers into *B. subtilis* recipients. The recipient strains were grown to competence in competence medium (CM) at 37 °C [46] for 6 h and then approximately 1 µg of DNA added to 500 µL of competent cells. Transformants were selected on LB agar supplemented with a specific antibiotic (chloramphenicol (Cm) 10 µg/mL, kanamycin (Kan) 50 µg/mL, or spectinomycin (Spec) 100 µg/mL) at 37 °C. Competence in strains with *comQ* deletion was induced by the addition of exogenous ComX, as previously described [47]. Integration of P*_epsA_*-*gfp* reporter fusion into different strains was performed by transformation with YC164 genomic DNA [37] (Table 1). The *srfA::Tn917* (*mls*; lincomycin 12.5 µg/mL and erythromycin 0.5 µg/mL) mutant was prepared by transformation with BM1044 genomic DNA [48] (Table 1). Strains with P*_tapA_*-*yfp* reporter fusion were constructed by transformation with BM1115 genomic DNA [49]. To prepare strains carrying the P*_spoIIQ_*-*yfp* reporter fusion, the plasmid pKM3 [50] was transformed into indicated recipients (Table 1). We transformed strains with the plasmid pMS17 with the kanamycin resistance marker, and the plasmid pMS7 with the chloramphenicol resistance marker to construct P*_43_*-*mKate2* reporter fusion strains (Table 2). To prepare strains with the P*_srfAA_*-*yfp* reporter fusion, DL722 chromosomal DNA was transformed into different strains [24]. To construct the NCIB 3610 QS mutant, the plasmid pMiniMAD2-updowncomQ (Table 2) was used [47]. The plasmid was transformed into *B. subtilis* NCIB 3610 *comI^Q12L^* as previously described [47,51].

The P*_43_*-*yfp* construct from the Pkm3-p43-*yfp* plasmid [49] was digested with EcoRI and BamHI and ligated into the pSac-Cm [52] plasmid to form the pEM1071 plasmid carrying a P*_43_*-*yfp* fusion inside the *sacA* integration site. P*_43_* is a constitutively expressed promoter in *Bacillus subtilis* [53] located upstream from the *yfp* gene in the pEM1071 plasmid. This vector was then digested with HindIII and BamHI restriction enzymes to remove the *yfp* gene from the original vector and then simultaneously ligated with the digested *mKate2* fragment to construct the plasmid pMS7. The *mKate2* sequence was PCR amplified using the P3F and P3R primer pair (Table 3) and the genomic DNA of BM1097 as the template [54]. To construct the plasmid pMS17, the plasmid pMS7 was digested with EcoRI and BamHI restriction enzymes, and the digested P*_43_*-*mKate2* fragment was ligated into pSac-Kan EcoRI/BamHI restriction sites [52].

### 2.2. Growth Conditions

Bacterial overnight cultures were inoculated directly from glycerol stocks at −80 °C grown in LB medium with the appropriate antibiotics at 37 °C with shaking at 200 rpm. Pellicle biofilms were grown by re-suspending an overnight culture (1% *v*/*v*) in liquid MSgg medium, (5 mM potassium phosphate tribasic (pH 7), 100 mM MOPS (pH 7), 2 mM MgCl_2_, 700 mM CaCl_2_, 50 mM MnCl_2_, 50 mM FeCl_3_, 1 mM ZnCl_2_, 2 mM thiamine, 0.5% glycerol, 0.5% glutamate, 50 mg/L tryptophan, 50 mg/L phenylalanine) [31], and incubating the culture in static conditions at 37 °C for up to 40 h. The heterologously expressed ComX in M9 spent medium was prepared as described previously [47]. Briefly, *E. coli* ED367 [57] was used as a heterologous expression strain where the transcription of the *comQ* and *comX* genes was induced with isopropyl β-D-1-thiogalactopyranoside (IPTG). After induction with IPTG, the ED367 filtered spent medium with ComX was added to the liquid MSgg medium (20% *v*/*v*). *E. coli* ED367 spent medium, where *comQX* expression was not induced by IPTG, was used as a control.

### 2.3. Biochemical Composition of Extracellular Polymers and CFU Counts Determination in Pellicle Biofilms

Pellicle biofilms were grown in 20 mL of liquid MSgg medium at 37 °C for up to 40 h in Petri dishes (90 mm diameter). At indicated times, pellicle biofilms were collected and transferred into two centrifuge tubes containing 1 mL of physiological saline solution. Pellicle biofilms were kept on ice during sonication with the MSE 150 Watt Ultrasonic Disintegrator Mk2 at three 5-s bursts and amplitude of 15 µm. After disintegration, cell counts were determined in the pellicle biofilm by colony forming units (CFU) on LB agar after 24 h of incubation at 37 °C. Spores were enumerated as CFUs/mL after heating cell suspensions to 80 °C for 30 min. The spore fraction was determined by dividing heat resistant CFUs with total CFUs. Extracellular polymers were extracted from pellicle biofilms, as described previously [29]. The total sugar content in the extracted polymer fraction was determined by the phenol–sulfuric acid method as described before [58] and the total protein content by the Bradford protein assay [59].

### 2.4. Spent Media Droplet Surface Wetting Assay

Pellicle biofilm spent media were collected after 40 h of static growth in MSgg medium at 37 °C, two-fold serially diluted in distilled water, and surfactin concentrations were estimated visually by comparing 20 µL droplets placed on a parafilm strip. As the increase in the wetting surface area of the droplet increases with surfactant concentration, we estimated relative surfactin concentration in the spent medium based on the surface area. Distilled water droplets served as a control.

### 2.5. Pellicle Biofilm Bulk Fluorescence Measurements

Pellicle biofilms were grown in 200 µL MSgg medium in sterile 96-well black transparent bottom microtiter plate. If needed, and as indicated, strains were supplemented with ComX. Microtiter plates were incubated in the Cytation 3 imaging reader (BioTek Instruments, Inc., Winooski, VT, USA) at 37 °C without shaking. Optical density at 650 nm and fluorescence intensity were measured in half hour intervals for up to 60 h. The gain settings, excitation, and emission wavelengths for every transcriptional reporter are shown in Table S1. Each strain was tested in three independent replicates at specified times. Results represent the averages of all the wells with 95% confidence intervals.

Isogenic strains without the fluorescent reporter (background strains) were always cultured in the same microtiter plate as the experimental strains to estimate the background fluorescence. To calculate the final fluorescence of the experimental strain, the average fluorescence intensity of its background strain (grown in eight wells) was deducted from the fluorescence intensity of the experimental strain at each time point.

### 2.6. Pellicle Biofilm Morphology, Hydrophobicity Estimation and Confocal Laser Scanning Microscopy

Pellicle biofilms were grown in 2 mL of MSgg medium in 35-mm diameter glass-bottom Petri dishes and imaged with the Leica WILD M10 stereomicroscope. Pellicle biofilm hydrophobicity was evaluated by spotting a 50 µL droplet of 0.5% (*w*/*v*) methylene blue solution in the center of the pellicle biofilm and the diffusion of the dye recorded over time by a video camera and then edited by Lightworks v. 14.5 trial video editing software. Cells in pellicle biofilms were visualized by confocal laser scanning microscope after removing the spent medium from underneath the pellicle biofilm by careful pipetting. We used the 100× immersion objective (NA 0.4) and roughly determined the bottom of the pellicle to set up a Z-stack. Pellicle biofilms carrying transcriptional reporters fused to specific promoters or/and constitutive promoters were then visualized using an LSM 800 microscope (Zeiss, Göttingen, Germany). Excitation laser wavelength, emission filter setup, and other laser settings are shown in Table S2. Averaging was set at eight, scanning was unidirectional, pixel time equaled 0.58 μs, and the frame time was 9.11 s.

Fluorescence images were taken with an AxioCam MRm Rev.3 camera. The captured images were analyzed using Zen Blue v. 2.3 lite software (Zeiss, Göttingen, Germany). Pixel intensity values were normalized using the best fit function. Images are presented as orthogonal displays with the Z-stack image that exhibited the highest fluorescence intensity of either the P*_epsA_*-*gfp* P*_tapA_*-*yfp* or P*_spoIIQ_*-*yfp* channels.

### 2.7. Flow Cytometry

Cultures were inoculated with 1% (*v*/*v*) of an overnight LB culture and grown at 37 °C in 4 mL of MSgg medium in 12-well microtiter plates without shaking. Pellicles were partially homogenized with a 5 mL pipette tip and transferred into a new container. The pellicle was partially disrupted by sonication with a 30-s burst at an amplitude of 15 µm. Afterward, the disrupted pellicle was treated with 4% (*w*/*v*) paraformaldehyde for 7 min at room temperature and washed in GTE buffer (glucose 50 mM, ethylenediaminetetraacetic acid (EDTA) 10 mM, Tris-HCl 20 mM, pH 8 ± 0.2). Cells were stored at room temperature until further analysis. The disrupted pellicle biofilms were suspended in 1 mL phosphate-buffered saline (PBS) containing 0.5% (*w*/*v*) bovine serum albumin. The cell suspensions were diluted 1:1000 into filtered GTE buffer containing 1 µg/mL 4′,6-diamidino-2-phenylindole (DAPI) and analyzed using a LSR Fortessa cytometer (BD Biosciences, Franklin Lakes, NJ, USA). Forward and side scatter, DAPI (355 nm laser, 450 nm/50 nm bandpass filter), and green fluorescent protein/yellow fluorescent protein (GFP/YFP) (488 nm laser, 530 nm/30 nm bandpass filter) were detected with photomultiplier voltages between 300 and 500 V. GFP and YFP signals were measured for 30,000 DAPI-gated events per sample. Data were analyzed using FlowJo 10 with samples gated on the upper limit of signal from fluorophore-free cells to define the GFP/YFP negative/positive cells in the population analyzed.

### 2.8. Statistical Analysis

All experiments were performed in at least three time-independent biological replicates. In the experiments concerning bulk pellicle fluorescence measurements in the microplate reader, we subtracted the estimate for background fluorescence intensity (averaged from all the wells with strains without fluorescent transcriptional reporter fusions) from every microtiter plate as well as fluorescence measurements. Afterward, fluorescence intensity in each well was averaged and a 95% confidence interval calculated from all of the wells. For biochemical tests, flow cytometry tests, and CFUs, the averages of three independent biological replicates are shown with the standard error of means (SEM).

In all experiments, two groups of samples were compared by calculating a Student’s *t*-test and a one-way non-parametric Mann–Whitney U test. We treated the two groups of samples as being statistically different if both tests showed a *p*-value < 0.05.

## 3. Results

### 3.1. The Quorum Sensing ComQ Mutant Forms Thicker Pellicles than the Wild Type

**Figure 1:**
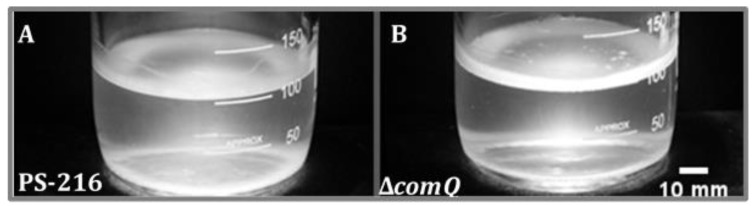
Pellicle biofilms of the *Bacillus subtilis* wild type PS-216 (**A**) and the ComX deficient PS-216 Δ*comQ* strain (BM1127) (**B**) grown statically in MSgg medium at 37 °C and in 250 mL bottles for 16 h.

To investigate the role of ComX in pellicle biofilm formation, we grew the wild type and the ComX deficient PS-216 mutant (Δ*comQ*) in MSgg medium [30]. We found that the Δ*comQ* mutant formed a prominent pellicle biofilm, which appeared thicker than the parental strain (Figure 1).

This observation contrasted with the generally held view that the ComQXPA QS system positively regulates biofilm formation due to its requirement for surfactin synthesis [23]. Therefore, we tested surfactin synthesis in both the wild type PS-216 [55] (hereafter PS-216) and the Δ*comQ* mutant (BM1127) strains. As expected, surfactin synthesis was approximately eight-fold lower in the Δ*comQ* mutant compared to PS-216 (Figure S1). The BM1044 mutant with an inactivated *srf* operon was used as a negative control. These results suggest that either surfactin is not essential for pellicle biofilm development in the PS-216 strain or other unknown factors may contribute to the proficient biofilm phenotype of the ComX deficient mutant.

Since *Bacillus* species can form highly hydrophobic biofilms [60,61], we assessed if the altered pellicle of the ComX mutants preserved this property. To estimate pellicle biofilm hydrophobicity, we spotted a 0.5% (*m*/*v*) methylene blue solution on the upper surface of the Δ*comQ* mutant and PS-216 pellicle biofilms. We observed that the droplet spread more slowly on the Δ*comQ* (BM1127) pellicle biofilm (Video S1), compared to PS-216, which suggests that the mutant pellicle is more hydrophobic and therefore structurally different to the PS-216 pellicle.

### 3.2. Expression of P_epsA_ and P_tapA_ Promoters Per Pellicle Is Higher in the QS Mutant than in the Wild Type Strain

**Figure 2:**
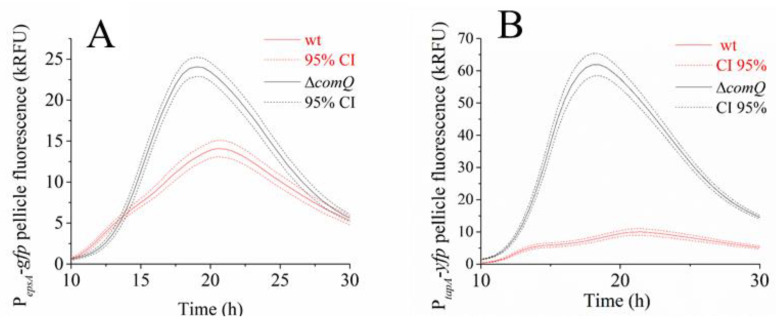
Bulk pellicle biofilm fluorescence measured in a microplate reader in *B. subtilis* PS-216 during static growth in MSgg medium at 37 °C. (**A**) Pellicle biofilm fluorescence intensity of the wild type phenotype P*_epsA_*-*gfp* reporter (wt; BM1631) strain and the Δ*comQ* mutant phenotype (BM1622) reporter strains. (**B**) Pellicle biofilm fluorescence intensity of the P*_tapA_*-*yfp* wild type phenotype (wt; BM1613) and Δ*comQ* phenotype (BM1614) reporter constructs. The line labelled CI shows the 95% confidence interval of the measurements.

The thicker pellicles formed by the peptide deficient mutant led us to question the postulated role of ComX in promoting biofilm formation. Therefore, we analyzed the activity of the P*_epsA_* promoter, which controls the expression of the *epsA-O* operon responsible for the synthesis of the major biofilm matrix polysaccharide. We also tested the P*_tapA_* promoter activity, which controls the synthesis of the TasA fibers [62] in the pellicles. We first monitored the activity of both promoters at the population level in the PS-216 strain (wt; BM1631) and Δ*comQ* (BM1622) strains carrying the P*_epsA_*-*gfp* reporter or in the BM1613 (wt) and BM1314 (Δ*comQ*) strains carrying the P*_tapA_*-*yfp* reporter, respectively. Both reporters are transcriptional fusions. To estimate fluorescence from both reporters in the entire pellicle biofilm, we measured the bulk pellicle fluorescence by fluorimetry. Our analysis revealed that both reporters exhibited a higher pellicle fluorescence intensity in the Δ*comQ* compared to the wild type (Figure 2A,B), which is inconsistent with ComX promoting overall biofilm formation.

The impact of the *comQ* mutation on pellicles formed by the *B. subtilis* NCIB 3610 strain was also tested. The PS-216 isolate, used here as the primary model strain, is very similar at the genomic sequence level to NCIB 3610, which is frequently used in *B. subtilis* biofilm research [55,63]. The NCIB 3610 isolate responded to ComX deficiency in a similar manner to the PS-216 isolate (Figure S2), albeit with some minor differences. The NCIB 3610 isolate produced comparatively less surfactin than PS-216, which further decreased in Δ*comQ* (Figure S1). It also responded to ComX deficiency similarly albeit less dramatically at the level of bulk P*_epsA_*-*gfp* expression than PS-216 derivative (Figure 2A, Figure S2). Although subtle strain specific differences between PS-216 and NCIB 3610 in biofilm formation exist, both strains respond comparably to ComX deficiency. This suggests that biofilm associated phenotypes observed in PS-216 may be more generally applicable.

### 3.3. The Quorum Sensing Mutant Pellicles Exhibit a Different Pellicle Morphology and Distributions of Cells with Active P_epsA_ and P_tapA_ Dependent Expression

**Figure 3:**
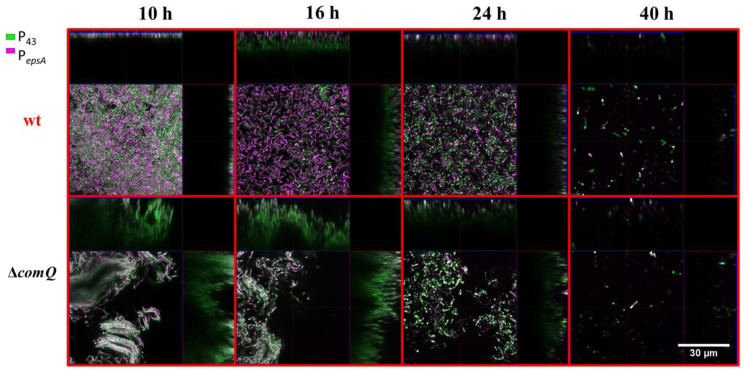
Pellicle biofilms of the PS-216 *Bacillus subtilis* wild type (wt; BM1631) and the QS mutant (Δ*comQ;* BM1622) phenotypes carrying P*_43_*-*mKate2* (false-colored green) and P*_epsA_*-*gfp* (false-colored magenta) transcriptional reporters during static growth in MSgg medium at 37 °C and visualized under a confocal microscope at the indicated time points.

**Figure 4:**
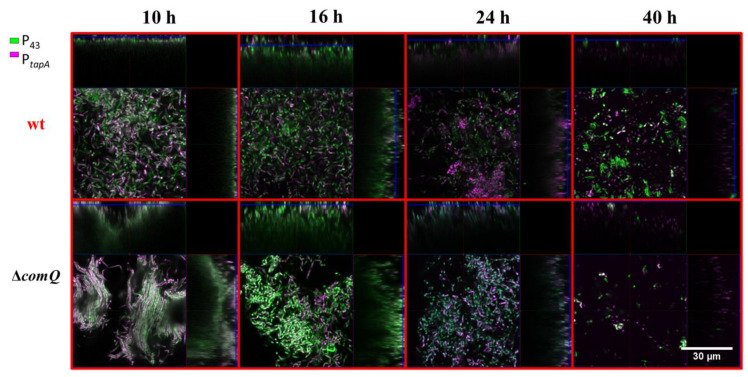
Pellicle biofilms of the PS-216 *Bacillus subtilis* wild type (wt; BM1613) and the QS mutant (Δ*comQ;* BM1614) phenotypes carrying P*_43_*-*mKate2* (false-colored green) and P*_tapA_*-y*fp* (false-colored magenta) transcriptional reporters during static growth in MSgg medium at 37 °C and visualized under a confocal microscope at the indicated time points.

In a biofilm, *B. subtilis* cells are subjected to spatiotemporal regulation of gene expression, which results in the coexistence of multiple cell types and a complex macroscale architecture in the mature community [64,65]. We lack an understanding of how ComX influences the spatiotemporal distribution of matrix producing cells in pellicle biofilms. To shed light on the influence exerted by ComX, we monitored the distribution of cells expressing the matrix genes using P*_epsA_-gfp* or P*_tapA_*-*yfp* reporters as markers, while detecting metabolically active cells using a P_43_-*mKate2* fluorescence reporter [53] carried by each strain. After removing the liquid media from the well at indicated time points, we directly visualized the pellicles by confocal microscopy. The Δ*comQ* pellicle biofilms lacking ComX had a different morphology from wild type, and the pellicles were 2.5-fold thicker with a depth of up to 30 µm compared with the wild type biofilms that had a depth of up to 12 µm (Figure 3 and Figure 4).

This analysis confirms our initial visual assessment of the pellicle biofilm (Figure 1). We further noted that the wild type cells in the pellicle were densely packed and evenly distributed at the 10 h time point. In contrast, the cells in the Δ*comQ* biofilm were less densely packed than those within the wild type pellicles. The Δ*comQ* mutant formed bundles of long chains of cells at early time points (10 h): cell chaining is one of the hallmarks of the biofilm phenotype [66].

Confocal microscopy images (taken at 10 h and 16 h time points) suggest that the wild type biofilms have a higher proportion of cells with the active P*_epsA_* promoter than the mutant biofilms, which is consistent with the published results [23]. Mostly, the P*_epsA_*activated promoter cells occupied the bottom of the pellicle (Figure 3). In contrast, we did not observe a substantial difference in the activity of the P*_tapA_* promoter between the two strains, the wild type (BM1631), and Δ*comQ* (BM1622) for any of the examined time points (Figure 4). By 40 h, we detected only a few P_43_ fluorescing (false-colored green) cells in both the wild type and *comQ* biofilms, which now appeared similar in morphology. Although the weak fluorescence from the P_43_-*mKate2* fluorescence reporter suggested their low metabolic activity, the biofilms were still visible by the naked eye, demonstrating that the biomass was not dispersed. In total, the confocal microscopy confirms that ComX during early stages of biofilm development shapes the thickness and morphology of this multicellular structure. It also supports a positive effect of ComX on the P*_epsA_*, but not on P*_tapA_*promoter.

### 3.4. P_tapA_ But Not P_epsA_ Dependent Expression Is More Active and in a More Substantial Portion of Cells in the ΔcomQ Mutant than in the Wild Type Strain

The microscopic imaging of the whole pellicle indicated heterogeneous activation of P*_epsA_* and P*_tapA_*. However, the microscopy of the undisturbed pellicle only provided qualitative results of the distribution of cell types. To obtain quantitative information at a single-cell resolution, we opted to follow the activity of these two promoters by flow cytometry. The wild type and ComX deficient (Δ*comQ*) strains were grown at 37 °C in 4 mL of MSgg medium in 12-well microtiter plates without shaking for the stated times. We disrupted the pellicle at 16 and 24 h time points and employed flow cytometry on the cell suspension to determine the percentage of cells that express the *epsA* and *tapA* operons. These experiments calculate the relative percentage of cells expressing each operon and the level of transcription at a single cell level.

**Figure 5:**
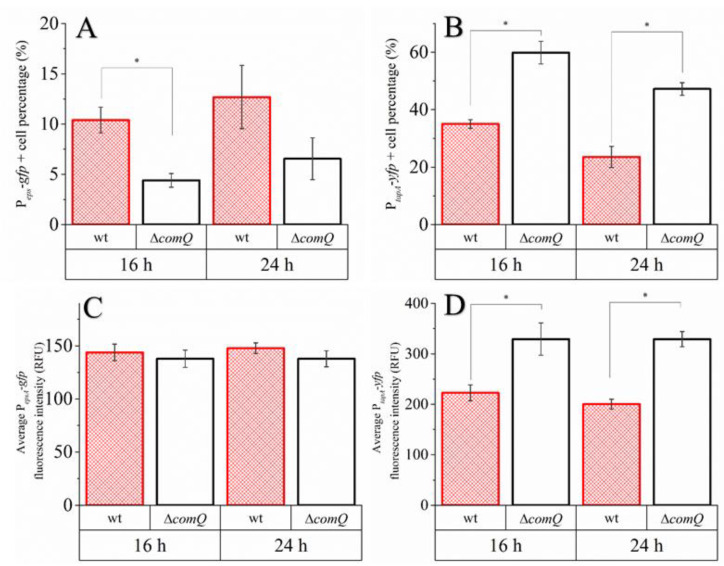
Comparisons of single-cell P*_epsA_* (wt; BM1103 and Δ*comQ*; BM1458) and P*_tapA_* (wt; BM1115 and Δ*comQ* BM1126) promoter activity in *B. subtilis* PS-216 during static growth in MSgg medium at 37 °C. (**A**) Percentage (relative to total cell count) of wild type phenotype (wt) and QS mutant (Δ*comQ*) cells expressing from P*_epsA_*. (**B**) Percentage (relative to total cell count) of cells expressing from P*_tapA_*. (**C**) Average fluorescence intensity of cells expressing from P*_epsA_*. (**D**) Average fluorescence intensity of cells expressing from P*_tapA_*. Data points represent averages with the standard error of means (SEM) of three biological replicates. Statistical significance (marked with *) was determined using a one-way Mann–Whitney test and a Student’s *t*-test (*p* < 0.05).

The results presented in Figure 5A show that only 10.4% ± 1.3% of cells express the P*_epsA_-gfp* reporter and the percentage (relative to the total cell count) of cells with P*_epsA_* promoter guided transcription being higher in the wild type (BM1103) than in the mutant (4.4% ± 0.7%; BM1458) at the 16 h time point. Although still detectable, this difference is not statistically significant at the 24 h time-point, where 12.7% ± 3.1% of wild type and only 6.5% ± 2.0% of Δ*comQ* cells express the P*_epsA_* regulated fluorescent reporter (Figure 5A). The percentage of cells that express the P*_tapA_-yfp* reporter in the wild type phenotype (BM1115) equaled to 35.0% ± 1.5% of cells at 16 h and 23.5% ± 3.6% of cells at 24 h. At both time points, the *comQ* pellicle (BM1126) harbored a significantly higher percentage of fluorescent cells (59.8% ± 3.9% at 16 h and 47.2% ± 2.1% at 24 h) (Figure 5B). There were no statistically significant differences detected in terms of average single cell P*_epsA_* expression (Figure 5B). The average intensity of P*_epsA_* expressing cells in the wild type reporter (BM1103) equaled 144 RFU ± 8 RFU at 16 h and 148 RFU ± 5 RFU at 24 h. The average intensity of P*_epsA_* expressing cells in the Δ*comQ* reporter (BM1458) was similar, and equaled 138 RFU ± 8 RFU at 16 h and 138 RFU ± 8 RFU at 24 h (Figure 5C). We did, however, detect a statistically significant difference between the wild type and the mutant expression profiles when we measured the average P*_tapA_* activity per cell. The average intensity of P*_tapA_* expressing cells in the wild type reporter (BM1115) equaled 223 RFU ± 16 RFU at 16 h and 200 RFU ± 10 RFU at 24 h. The average intensity of P*_tapA_* expressing cells in the Δ*comQ* reporter (BM1126) on the other hand, equaled 329 RFU ± 32 RFU at 16 h and 329 RFU ± 15 RFU at 24 h (Figure 5D).

In conclusion, the ComQX QS system increases the fraction of cells expressing the *epsA* operon during early biofilm development without changing the average intensity of P*_epsA_* guided transcription. In contrast, ComX decreases the average activity and fraction of cells with P*_tapA_* activity.

### 3.5. Extracellular Polymer Extracts of QS Mutant Pellicles Contain More Sugar and Protein

Our results show that ComX negatively affected P*_tapA_* transcription, but positively guided P*_epsA_* transcription. Therefore, it was essential to verify whether ComX deficient mutants preserve this pattern at the level of production of matrix components. To estimate the overall monosaccharide and protein content in the biofilm matrix, we extracted the extracellular polymers from the wild type (PS-216) and Δ*comQ* (BM1127) pellicles using a simplified method of Dogsa et al. [29]. We then quantified the amount of monosaccharide and protein in the extracellular polymer extracts by using the reducing sugar assay [58] and the Bradford protein assay [59].

**Figure 6:**
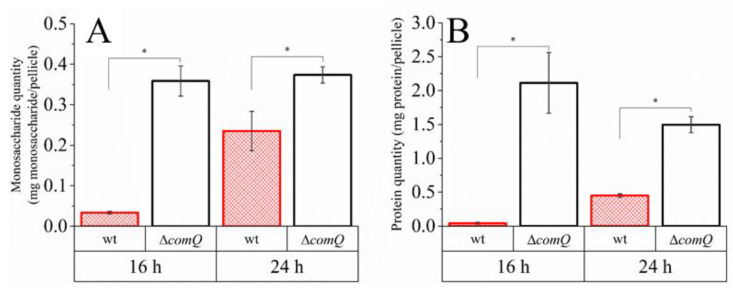
Monosaccharide and protein quantity in *B. subtilis* PS-216 (wt; PS-216) and QS mutant phenotype (ΔcomQ; BM1127) pellicles during static growth in the MSgg medium at 37 °C. (**A**) Monosaccharide content was determined with the phenol–sulfuric acid method. (**B**) Protein content was determined with the Bradford protein assay. Averages with the standard error of means (SEM) of three biological replicates are shown. Statistical significance (marked with *) was determined using a one-way Mann–Whitney test and a Student’s *t*-test (*p* < 0.05).

Despite our finding that a lower percentage of cells actively expressed the P*_epsA_* promoter in the *comQ* strain using flow cytometry, the overall monosaccharide content did not follow this pattern. Polymer extracts of the Δ*comQ* biofilms harbored a higher amount of monosaccharide per pellicle than the wild type extracts (Student’s *t*-test and one-way Mann–Whitney U test; *p* < 0.05) at 16 h (10-fold more) and 24 h (1.6-fold more) (Figure 6A).

Therefore, the synthesis of matrix components followed the activity pattern of the P*_tapA_*, but not of the P*_epsA_* promoter.

### 3.6. The QS Deficient Mutant Has Lower Spore Counts during the Early Stages of Biofilm Development

**Figure 7:**
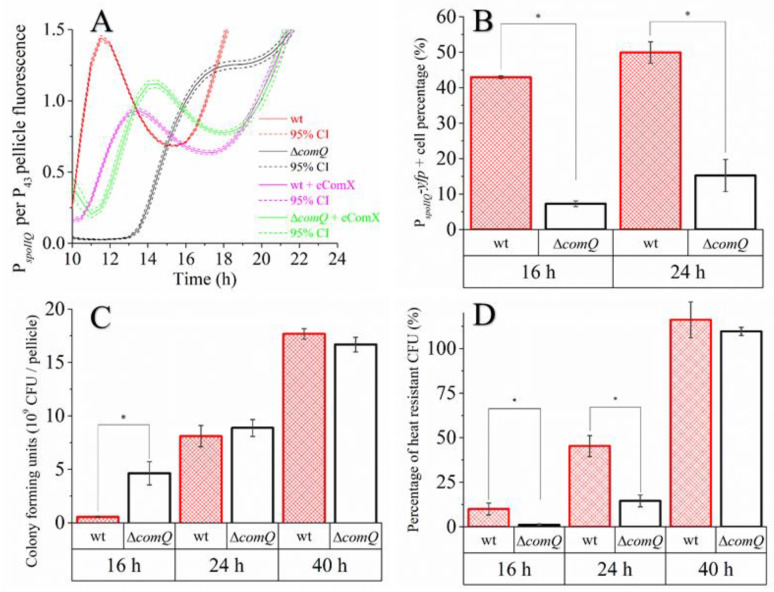
Comparisons of *B. subtilis* PS-216 wild type (wt) and QS mutant (Δ*comQ*) during static growth in MSgg medium at 37 °C. (**A**) Pellicle biofilm P*_spoIIQ_-yfp* fluorescence normalized per P_43_-mKate2 fluorescence with and without exogenous heterologously expressed ComX. (**B**) The fraction of cells with an active P*_spoIIQ_*-*yfp* promoter per all cells analyzed by flow cytometry. (**C**) Total pellicle biofilm CFU counts. (**D**) The fraction of the heat resistant CFU per the total non-heat-treated pellicle CFU. Averages with the standard error of means (SEM) of three biological replicates are shown. Statistical significance (marked with *) was determined using a one-way Mann–Whitney test and a Student’s *t*-test (*p* < 0.05), and by calculating the 95% confidence interval on panel B (dashed line).

Matrix transcribing cells transition into spore forming cells during the time course of biofilm formation [67]. Thus, we next tested whether ComX influences the dynamics of sporulation during biofilm development. To do this, we examined the impact of ComX on the expression of the sporulation marker gene, *spoIIQ*. First, we measured the P*_spoIIQ_-yfp* dependent bulk fluorescence by fluorometry over time and normalized it by P_43_ (constitutive promoter) dependent bulk fluorescence to roughly account for culture growth (Figure 7A). Normalized fluorescence was found to be higher in the wild type (BM1625) than in the mutant (BM1626) during the first 10–14 h of incubation. Around 14 h of incubation, the normalized P*_spoIIQ_*-*yfp* fluorescence increased rapidly, reaching the fluorescence of the wild type. When we added heterologous ComX to the Δ*comQ* mutant (BM1626) at the beginning of the incubation, its expression profile became comparable to when heterologous ComX was added to the wild type strain (BM1625) (Figure 7A). The sole addition of heterologous ComX to the wt strain, however, also slightly modified its normalized expression profile. This is most likely because ComX was already added at the onset of growth. We next quantified the number of cells in the pellicle that express the *spoIIQ* gene by flow cytometry (Figure 7B) using the P*_spoIIQ_*-*yfp* labelled wild type strain (BM1128) and the Δ*comQ* strain (BM1129). These results reveal that the P*_spoIIQ_* promoter was active in 42.9% ± 0.4% (SEM; standard error of means) of the cells in wild type (BM1128) at 16 h and 49% ± 3.0% of cells at 24 h. In the ComX deficient mutant (BM1129), 7.2% ± 0.8% of cells activated this gene at 16 h and 15.2% ± 4.5% at 24 h.

We finally compared the number of colony-forming units (CFU) in the wild type (PS-216) and Δ*comQ* (BM1127) pellicles (Figure 7C). The total CFU counts were comparable at 24 h and 40 h, however, the Δ*comQ* mutant exhibited a significantly higher CFU count in the pellicle at the 16 h time-point (Figure 7C).

We also measured the quantity of heat resistant spores in the population (Figure 7D). The wild type pellicle contained 9.9% ± 3.3% (SEM) at 16 h and 45.3% ± 5.9% at 24 h heat resistant CFU per total pellicle CFU. The QS mutant pellicle contained 1.1% ± 0.4% and 14.5% ± 3% of heat resistant CFU per total CFU at 16 h and 24 h, respectively (Figure 7B). At 16 h, the wild type pellicle therefore contained approximately 5-fold and at 24 h 3-fold more heat resistant CFUs than the Δ*comQ* mutant. After 40 h, all the pellicle CFUs were heat resistant in both strains (Figure 7D).

**Figure 8:**
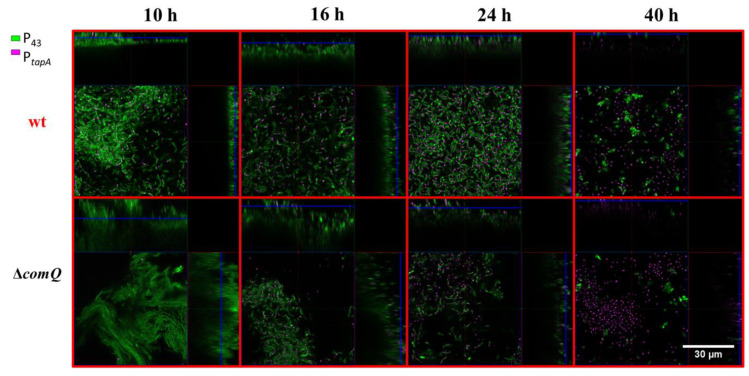
Confocal visualization of P*_spoIIQ_-yfp* (false-colored magenta) and P*_43_*-*mKate2* (false-colored green) expression in wild type (wt; BM1625) and QS mutant (Δ*comQ*; BM1626) pellicle biofilms during static growth in MSgg medium at 37 °C at the indicated time points.

Taken in combination, the difference in timing of the P*_spoIIQ_* promoter activity between the wild type and Δ*comQ* pellicle biofilm visualized by confocal microscopy (Figure 8) further supports that the wild type enters sporulation after only 10 h of incubation. In contrast, the Δ*comQ* pellicle biofilms did not show detectable P*_spoIIQ_-yfp* activity at this time.

Collectively, these results indicate that ComX deficiency decreases sporulation during the early stages of pellicle formation, a decrease that is most measurable at the 10 h time point where P*_spoIIQ_* activated cells are not detected by confocal microscopy in the Δ*comQ* mutant, but are visible in the wild type. We therefore conclude that ComX signals to the cell population to halt cell growth and production of matrix components. It has been recently shown that the main protein matrix component TasA also serves as an extracellular signal, which can trigger cell motility [68], therefore potentially enabling cells to migrate and colonize new ecological niches. However, since ComX mediated quorum sensing appears to decrease TasA production, it appears that QS promotes an increased investment into spore development instead of cell motility.

## 4. Discussion

The ComQXPA QS system upregulates surfactin production [22,57,69], which promotes transcription of the *epsA-O* operon [23]. Hence, a generally accepted view is that ComX promotes biofilm formation. However, our results showed that the ComX deficient mutant still developed prominent and structurally even more prominent biofilms than the wild type strain. The QS mutant biofilms expressed the P*_tapA_* promoter in a higher proportion of cells and accumulated a greater quantity of proteins and sugars than the wild type biofilms. In contrast, ComX deficient mutants produced fewer spores during early stages of biofilm development. These results indicate that ComX dependent QS negatively affects investment into multicellularity and positively to commitment to spore formation.

The average expression level from the *tapA* promoter and the percentage of cells expressing *tapA* were both higher in the QS mutant cells (Figure 5B,D), which indicates that ComX negatively regulates the synthesis of matrix proteins, but not the P*_epsA_* activity. Although, earlier work suggests that *epsA-O* and *tapA* operons are co-regulated [67,70], recently published results [71] and our observations do not support their tight co-regulation. The effect on P*_tapA_* and the absence of an effect on P*_epsA_* may be related to differential affinities of these promoters for SinR or AbrB as the concentration of Spo0A-P gradually increases due to the surfactin effect on phosphorelay. We also found ComX deficient mutants formed more elongated and chained cells (Figure 3, Figure 4, and Figure 8) compared to the wild type strain. Chained cells, a hallmark of *B. subtilis* biofilm formation, express P*_tapA_* regulated genes, while properly segmented cells have increased *epsA-O* transcription [71]. Therefore, we speculate that a yet unknown but ComX dependent mechanism inhibits the P*_tapA_* activity in pellicles and decreases the synthesis of biofilm matrix protein. Although ComX does not negatively influence P*_epsA_* activity (Figure 5A,C), the mutant biofilm matrix still accumulates more monosaccharides, which are indicative of a higher concentration of polysaccharides (Figure 6A,B). At this point, we cannot explain the molecular basis of this observation, but only speculate that the synthesis of matrix polysaccharides might be regulated by additional mechanisms that are under the influence of ComX and are independent of P*_epsA_* promoter activity.

Moreover, other signaling pathways like PhrC (CSF) and its cognate RapC phosphatase, which are involved in surfactin production through ComA-P [26], albeit with a lesser effect than ComX [72], may contribute to the synthesis of matrix components and biofilm development. The phosphorelay, which regulates accumulation of Spo0A-P, integrates a variety of signaling pathways including kinases, phosphatase, and additional Phr-Rap pairs [65]. However, future work will show whether ComX dependent regulation of biofilm development acts primarily through surfactin, the DegQ-DegS-DegU regulatory system [42], or the Spo0A phosphorelay [67].

Our results are in accordance with recent findings, indicating that surfactin is not generally essential for the formation of pellicle biofilms [73] with surfactin mutants exhibiting similar shifts in the expression of *epsA* and *tapA* operons, as we show here for the QS mutant. However, the results presented here suggest that the main regulatory path of biofilm development may include ComX and only subsequently surfactin.

By analyzing single-cell expression, we confirmed that ComX positively regulates the *epsA-O* operon [23]. We also quantified heat resistant spores, the percentage of the P*_SpoIIQ_-yfp* active cells by flow cytometry, and the P*_SpoIIQ_-yfp* bulk fluorescence during biofilm growth. We found that ComX promotes sporulation in a subpopulation of cells during the early stages of biofilm growth. Bacterial spores stop growing and are metabolically inactive [74]. Although we determined the lower proportion of cells with the P*_epsA_* active promoter, the absence of spores early in the ComX mutant may contribute to higher absolute numbers of matrix producers and higher sugar content due to prolonged growth. This result is in accordance with the idea that the phosphorylation of the Spo0A gradually increases [75]. An intermediate level of Spo0A-P triggers the transcription of the *epsA-O* operon, while a high level of Spo0A-P triggers sporulation [36]. This is evidenced by the delayed and more synchronous sporulation of the QS mutant (Figure 7). We speculate that low levels of Spo0A-P persist longer in the ComX deficient mutant due to low levels of surfactin, which adds to the activation of Spo0A-P in the wild type [23]. It is possible that other Phr-Rap pairs may substitute for surfactin signaling, but these act a bit late during the stationary phase [65,76]. Hence, without ComX to detect the increase in cell density and approaching starvation early on, cells produce polysaccharides rather than spores.

We here show that ComX deficient mutants have delayed sporulation in biofilms compared to the wild type, so ComX acts as a positive signal for sporulation. Recent work demonstrates that the timing of sporulation entry has consequences for spore quality, with early spore formers showing more efficient germination than late spore formers [77]. In addition to this delay, we also observed that the ComX defective mutant induced *spoIIQ* more synchronously than the wild type, which shows prominent heterogeneity in the expression of this marker gene. Phenotypic heterogeneity arises from several different intrinsic and extrinsic factors [78]. Sporulation in *B. subtilis* is subject to phenotypic heterogeneity due to the noise in the expression of transcriptional regulators, the phosphate transfer in the phosphorylation regulation cascades, and positive and negative feedback loop mechanisms [79]. There are several potential input signals, which may contribute to heterogeneous sporulation [80] and the division of labor in biofilms [81]. How a QS system in general and ComX in particular mechanistically contribute to the heterogeneity of gene expression is not yet understood [45]. We speculate that ComX informs the population on its growth rate and may act as a gatekeeper, which allows investments into late growth adaptations only when growth slows down. Given this, ComX deficiency will enable the population to skip this safety valve and overinvest into growth and matrix production rather than prepare for dormancy. However, ComX omission extends growth only for a short period. Soon, other signaling systems may attain their thresholds and inform the population of the famine and stress and override ComX deficiency.

*B. subtilis* is known for its bet hedging behavior in terms of spore formation, where a sub-population of cells initiates sporulation stochastically, regardless of the sensed external environmental stimuli (e.g., nutrient starvation) [13]. In a wild type population, the endospore forming process already start at the onset of floating biofilm formation. The bacterial population with an inactivated QS, however, did not exhibit such behavior, but invested into spore formation later. This suggests that the ComQXPA QS system serves as a signal that down modulates investment into growth and assures early investment into sporulation. This, on one hand, may enable bet hedging behavior, but on the other hand, may burden cells with the metabolic costs of early sporulation initiation.

## 5. Conclusions

This work shows that ComX inhibits matrix production, especially the TasA protein, and promotes sporulation during early stages of biofilm development in the wild type. Hence, we propose that this QS signaling system fine-tunes commitment to different biological states and therefore contributes to the phenotypic heterogeneity of *B. subtilis* biofilms.

## Figures and Tables

**Table 1:** Bacterial strains used in this study.

Strain Name	Background	Genome Description	Reference
***Bacillus subtilis* strains**			
PS-216	PS-216	wt	[55]
BM1127	PS-216	Δ*comQ*	[47]
BM1128	PS-216	*amyE::*P*_spoIIQ_-yfp* (Sp)	this work
BM1129	PS-216	Δ*comQ* *amyE::*P*_spoIIQ_-yfp* (Sp)	this work
BM1625	PS-216	*amyE::*P*_spoIIQ_-yfp* (Sp) *sacA::*P*_43_-mKate2* (Cm)	this work
BM1626	PS-216	Δ*comQ* *amyE::*P*_spoIIQ_-yfp* (Sp) *sacA::*P*_43_-mKate2* (Cm)	this work
BM1613	PS-216	*amyE::*P*_tapA_-yfp* (Sp) *sacA::*P*_43_-mKate2* (Cm)	this work
BM1614	PS-216	Δ*comQ* *amyE*::P*_tapA_*-*yfp* (Sp) *sacA*::P*_43_*-*mKate2* (Cm)	this work
BM1629	PS-216	*sacA::*P*_43_-mKate2* (Kan)	this work
BM1630	PS-216	Δ*comQ* *sacA::*P*_43_-mKate2* (Kan)	this work
BM1631	PS-216	*amyE::*P*_epsA_-gfp* (Cm) *sacA::*P*_43_-mKate2* (Kan)	this work
BM1622	PS-216	Δ*comQ* *amyE::*P*_epsA_-gfp* (Cm) *sacA::*P*_43_-mKate2* (Kan)	this work
DK1042	NCIB 3610	*comI^Q12L^*	[56]
BM1667	NCIB 3610	Δ*comQ* *comI^Q12L^*	this work
BM1623	NCIB 3610	*comI^Q12L^* *amyE*::P*_epsA_*-*gfp* (Cm)	this work
BM1624	NCIB 3610	*comI^Q12L^* Δ*comQ* *amyE*::P*_epsA_*-*gfp* (Cm)	this work
YC164	NCIB 3610	*amyE*::P*_epsA_*-*gfp* (Cm)	[37]
BM1103	PS-216	*amyE*::P*_epsA_*-*gfp* (Cm)	this work
BM1458	PS-216	Δ*comQ* *amyE*::P*_epsA_*-*gfp* (Cm)	this work
BM1115	PS-216	*amyE::P_tapA_-yfp* (Sp)	[49]
BM1126	PS-216	Δ*comQ* *amyE::P_tapA_-yfp* (Sp)	this work
BM1097	PS-216	*amyE*::P*_hyperspank_*-*mKate2* (Cm)	[51]
BM1044	PS-216	*srfA::Tn917* (mls)	[48]
BM1673	PS-216	Δ*comQ* *srfA::Tn917* (mls)	this work
DL722	3610	*amyE*::P*_srfAA_*-*yfp* (Sp)	[24]
BM1454	PS-216	*amyE*::P*_srfAA_*-*yfp* (Sp)	this work
BM1455	PS-216	Δ*comQ* *amyE*::P*_srfAA_*-*yfp* (Sp)	this work
***Escherichia coli strain***			
ED367	BL-21 (DE3)	pET22(b)—*comQ comX* from *B. subtilis* 168 (Amp)	[57]

**Table 2:** Plasmids used in this study.

Plasmid Name	Description	Reference
pKM3	*amyE::*P*_spoIIQ_*-*yfp* (Sp, Amp)	[50]
Pkm3-p43-yfp	*amyE::*P*_43_*-*yfp* (Sp, Amp)	[49]
pSac-Kan	*sacA::kan* (Amp)	[52]
pSac-Cm	*sacA::cat* (Amp)	[52]
pEM1071	*sacA*::P*_43_*-*yfp* (Cm, Amp)	this work
pMS7	*sacA::*P*_43_*-*mKate2* (Cm, Amp)	this work
pMS17	*sacA::*P*_43_*-*mKate2* (Kan, Amp)	this work
pMiniMAD2-updowncomQ	pMiniMAD2 with updown *comQ* between EcoRI and SalI sites (Mls, Amp)	[47]

**Table 3:** Oligonucleotides used in this study.

Oligonucleotide Name	Sequence 5′→3′	Reference
P3F	GTACAAGCTTAAGGAGGAACTACTATGGATTCAATAGAAAAGGTAAG	[54]
P3R	GTACGGATCCTTATCTGTGCCCCAGTTTGCT	[54]

## Supplementary Materials

The following are available online at https://www.mdpi.com/2076-2607/8/8/1131/s1, Figure S1: Semi quantification of surfactant concentrations in the pellicle biofilm spent media after 40 h of static growth in MSgg medium at 37 °C using a droplet surface wetting assay, Figure S2: Bulk pellicle fluorescence measurements of the P*_epsA_*-*gfp* reporter activity of *Bacillus subtilis* NCIB 3610 wild type (wt) and QS mutant (Δ*comQ*) phenotypes during static growth in MSgg medium at 37 °C. Averages of three biological replicates are shown. Statistical significance was determined by calculating the 95% confidence interval (dashed line); Table S1: Emission and excitation wavelengths and gain settings for transcriptional reporters used in this study, Table S2: Confocal laser scanning microscope settings for different transcriptional reporters used in this study; Video S1: Fast forward video showing diffusion of the 50 µL methylene blue droplet placed on the PS-216 wild type (wt) or QS mutant (Δ*comQ*) pellicle biofilms grown statically in MSgg medium at 37 °C for the time periods indicated on the video, https://www.youtube.com/watch?v=1FRfrhzIPKY&feature=youtu.be. Upload date: 17.9.2018.
