# Supplementary material for: The ComX Quorum Sensing Peptide of Bacillus subtilis Affects Biofilm Formation Negatively and Sporulation Positively
Source: Microorganisms. 2020 Jul 27;8(8):1131. doi: 10.3390/microorganisms8081131 (PMC7463575; doi:10.3390/microorganisms8081131)
Supplement: Supplementary file 1 [file microorganisms-08-01131-s001.pdf]

microorganisms

Supplementary Materials:

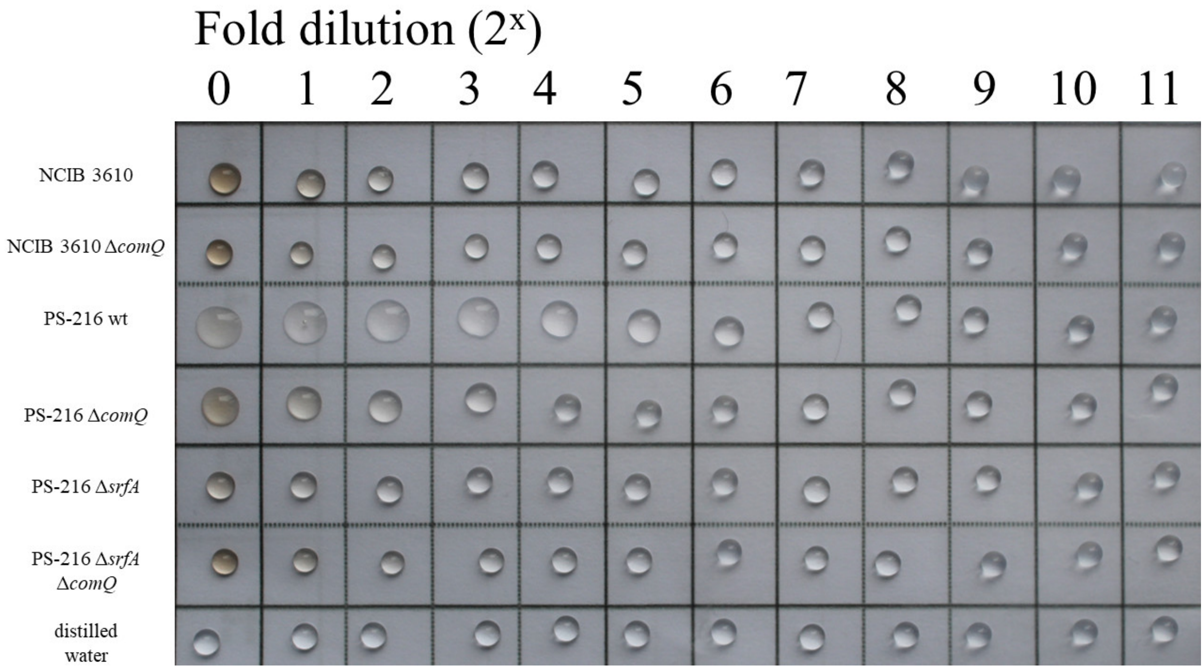

Figure S1: Semi quantification of surfactant concentrations in the pellicle biofilm spent media after 40 h of static growth in MSgg medium at 37 °C using a droplet surface wetting assay

.

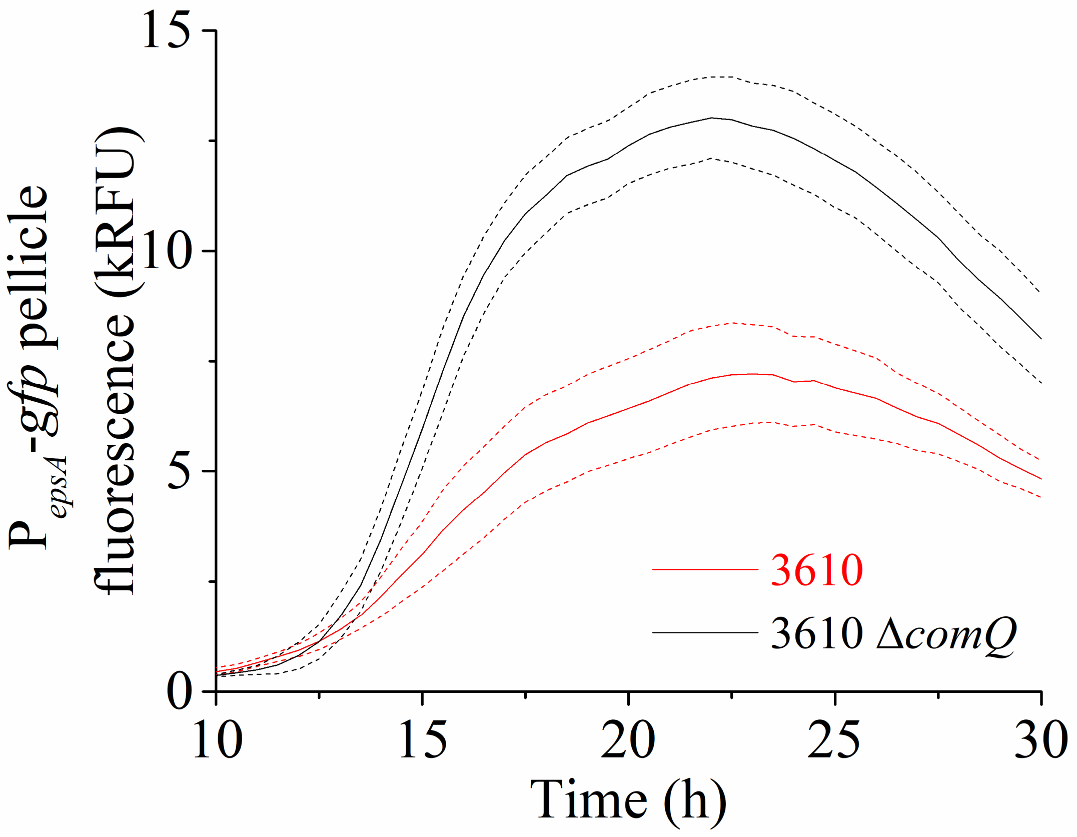

Figure S2. Bulk pellicle fluorescence measurements of the P$_{epsA}$-*gfp* reporter activity of *Bacillus subtilis* NCIB 3610 wild type (wt) and QS mutant (Δ*comQ*) phenotypes during static growth in MSgg medium at 37 °C. Averages of three biological replicates are shown. Statistical significance was determined by calculating the 95% confidence interval (dashed line);

Table S1

**Transcriptional reporter**	**Excitation wavelength [nm]**	**Emission wavelength [nm]**	**Gain**
P_{epsA}-*gfp*	480	510	100
P_{tapA}-*yfp*	500	530	80
P_{spoIIQ}-*yfp*	500	530	80
P_{43}-*mKate2*	570	620	100

Table S1: Emission and excitation wavelengths and gain settings for transcriptional reporters used in this study,

Table S2

**Transcriptional reporter**	**Excitation Laser wavelength [nm]**	**Emission filter [nm]**	**Laser power [%]**	**Master gain [v]**	**Pinhole [µm]**
**BM1631 and BM1622**					
P_{epsA}-*gfp*	**488**	**400-580**	**15**	**650**	**71**
P_{43}-*mKate2*	**561**	**585-700**	**2.5**	**600**	**84**
**BM1613 and BM1614**					
P_{tapA}-*yfp*	**488**	**400-590**	**7**	**600**	**72**
P_{43}-*mKate2*	**561**	**590-700**	**2.5**	**600**	**86**
**BM1625 and BM1626**					
P_{spoIIQ}-*yfp*	**488**	**400-590**	**7**	**600**	**72**
P_{43}-*mKate2*	**561**	**590-700**	**2.5**	**600**	**86**

Table S2: Confocal laser scanning microscope settings for different transcriptional reporters used in this study;

https://www.youtube.com/watch?v=1FRfrhzIPKY&feature=youtu.be

Video S1: Fast forward video showing diffusion of the 50 μL methylene blue droplet placed on the PS-216 wild type (wt) or QS mutant (Δ*comQ*) pellicle biofilms grown statically in MSgg medium at 37 °C for the time periods indicated on the video, https://www.youtube.com/watch?v=1FRfrhzIPKY&feature=youtu.be. Upload date: 17.9.2018.
